# Ultra-widefield retinal imaging for adjunctive resident training in retinal break detection

**DOI:** 10.1371/journal.pone.0253227

**Published:** 2021-06-23

**Authors:** I-Hung Lin, Chien-Cheng Chien, Yi-Hao Chen, Shu-i Pao, Jiann-Torng Chen, Ching-Long Chen

**Affiliations:** Department of Ophthalmology, Tri-Service General Hospital, National Defense Medical Center, Taipei, Taiwan; University of Warmia, POLAND

## Abstract

We evaluated the clinical utility of ultra-widefield imaging as an adjunctive tool for training resident ophthalmologists in the detection of retinal breaks. This was a prospective study conducted at a secondary health care center (Tri-Service General Hospital) in Taiwan. Participants were 44 patients (53 eyes) who were referred to our hospital after being diagnosed with retinal breaks. Patients first underwent an indirect ophthalmoscopy examination of the total fundus without scleral depression by our junior (first and second year) or senior (third and fourth year) resident ophthalmologist and then underwent an ultra-widefield imaging examination with a central image and four gaze-steered (up, down, nasal, and temporal) images to determine the number of retinal breaks in the total fundus and the four quadrants. Of the total 53 eyes, 31 were examined by junior residents and 22 were examined by senior residents. In the group of junior residents, ultra-widefield imaging was significantly better at detecting retinal breaks of the total fundus (49 vs. 33 retinal breaks, p < 0.001) and the temporal quadrant (17 vs. 10 retinal breaks, p = 0.018) than indirect ophthalmoscopy. In the group of senior residents, there was no significant difference in the ability to detect retinal breaks in the total fundus or each of the four quadrants with ultra-widefield imaging or indirect ophthalmoscopy. Our results indicate that, compared to indirect ophthalmoscopy, ultra-widefield imaging with a central image and four gaze-steered images has a better performance and is a useful adjunct tool for the detection of retinal breaks in junior resident training. Additionally, it could be a useful method for teaching indirect ophthalmoscopy examination to junior residents.

## Introduction

Retinal breaks are a well-recognized complication following separation of the cortical vitreous and posterior hyaloid membrane from the inner retina [1]. An estimated 6% of the general population have had a retinal break [2]. Retinal breaks that are left untreated can lead to retinal detachment in 30–50% of eyes [3] and cause vision loss [4]. Therefore, it is important to detect retinal breaks and provide prophylactic treatment to avoid progression to retinal detachment. The gold standard for diagnosis of retinal breaks is the use of the indirect ophthalmoscopy combined with scleral depression [5], whereas Shukla et al. have reported no difference in the peripheral vitreoretinal pathology using indirect ophthalmoscopy with or without scleral depression [6]. On literature review, the indirect ophthalmoscopy with scleral depression could provide a field of view of up to ora serrata [7, 8], which means that a field of view of 230° is visible [9]. However, in the clinic, the success rate of visualizing the lesion near ora serrata using indirect ophthalmoscopy, without scleral depression, depended on the experience of the examiner and proper positioning of the patient. Thus, the covered field of view on indirect ophthalmoscopy, without scleral depression, by ophthalmology residents might have been ≦230°. Moreover, performing indirect ophthalmoscopy is difficult for junior residents, partly because of the hand-eye coordination required but mainly because of the complication that the inverted image poses on spatial localization [10]. Therefore, many teaching methods and simulators have been designed to help junior residents better perform indirect ophthalmoscopy examinations [10, 11].

Besides indirect ophthalmoscopy, photographic imaging of the fundus can also be used to identify retinal breaks. Traditional color fundus photographic imaging can only provide a small angle, about a 30° to 45° field of view of the fundus [12]. Multiple images taken using traditional color fundus photography can be manually overlapped to create a montage; for example, seven standard 30° color fundus images may be combined to produce a 75° field of view [13]. However, even a 75° field of view only partially reveals the retina, which may not be sufficient to see retinal breaks at the peripheral sides. In recent years, ultra-widefield (UWF) retinal imaging has been developed, which allows a 105° to 200° field of view (depending on the UWF retinal imaging system used) when taking central images [14–16]. When four gaze-steered images (up, down, temporal, and nasal) are obtained along with central images in UWF retinal imaging examinations, a field of view of up to 220° means that 97% of the retina is visible [17]. peripheral retinal breaks can be visualized using UWF retinal imaging.

Because indirect ophthalmoscopy is difficult to perform for junior residents and UWF retinal imaging provides up to 97% visualization of the retina, this technique may be used as a teaching method to help junior residents to improve their performance of indirect ophthalmoscopy for the detection of retinal breaks. The purpose of this study was to compare the ability of UWF retinal imaging and indirect ophthalmoscopy to detect retinal breaks in examinations performed by junior and senior ophthalmologists. This allowed us to evaluate whether UWF imaging is a useful, adjunctive tool in the detection of retinal breaks, especially for junior residents, and whether it can be used as a method to teach indirect ophthalmoscopy to the aforementioned group.

## Materials and methods

This prospective observational study was performed at Tri-Service General Hospital, National Defense Medical Center, Taipei City, Taiwan, from April 1st, 2019 to September 30th, 2020. The study was conducted with the approval of the Institutional Review Board (Registration No. C202005046) and adhered to the tenets of the Declaration of Helsinki. All patients provided informed consent for participation in the study.

This study included patients with symptoms of floaters and/or photopsia who came to our clinic or the emergency department of the Tri-Service General Hospital, National Defense Medical Center, Taipei City, Taiwan during the study period and were diagnosed with a retinal break. Patients with a disease causing corneal opacity or vitreous opacity, such as cornea decompensation or vitreous hemorrhage, which cause poor image quality, were excluded. Patients who were not willing to take part in the UWF imaging or indirect ophthalmoscopy examination were also excluded. Finally, data from 53 eyes of 44 patients with retinal breaks were collected. Nine patients had retinal breaks in both eyes.

Patients first underwent an indirect ophthalmoscopy examination with pupil dilatation and without scleral depression of the total fundus, followed by UWF imaging examination with pupil dilatation, using Optos California UWF retinal imaging (Optos, Inc. Marlborough, MA, USA). These examinations were perforemed by junior ophthalmology residents (31 eyes of 25 patients) or senior ophthalmology residents (22 eyes of 19 patients). Indirect ophthalmoscopy examination was done using a binocular indirect ophthalmoscope (HEINE OMEGA 500 binocular indirect ophthalmoscope) with a 20D lens (Ocular MaxAC® 20D). Patients were randomized to be examined by either junior or senior residents. In Taiwan, ophthalmology residency training is a 4-year program. Junior residents were defined as residents in their first or second year of resident ophthalmologist training, and senior residents were defined as those in their third or fourth year of resident ophthalmologist training. Overall, 6 junior residents and 5 senior residents were included in this study. After the indirect ophthalmoscopy examination was performed by the junior or senior residents, the examination was repeated by one of two randomly assigned qualified attending ophthalmologists, Ching-Long Chen and Shu-I Pao.

**Fig 1:**
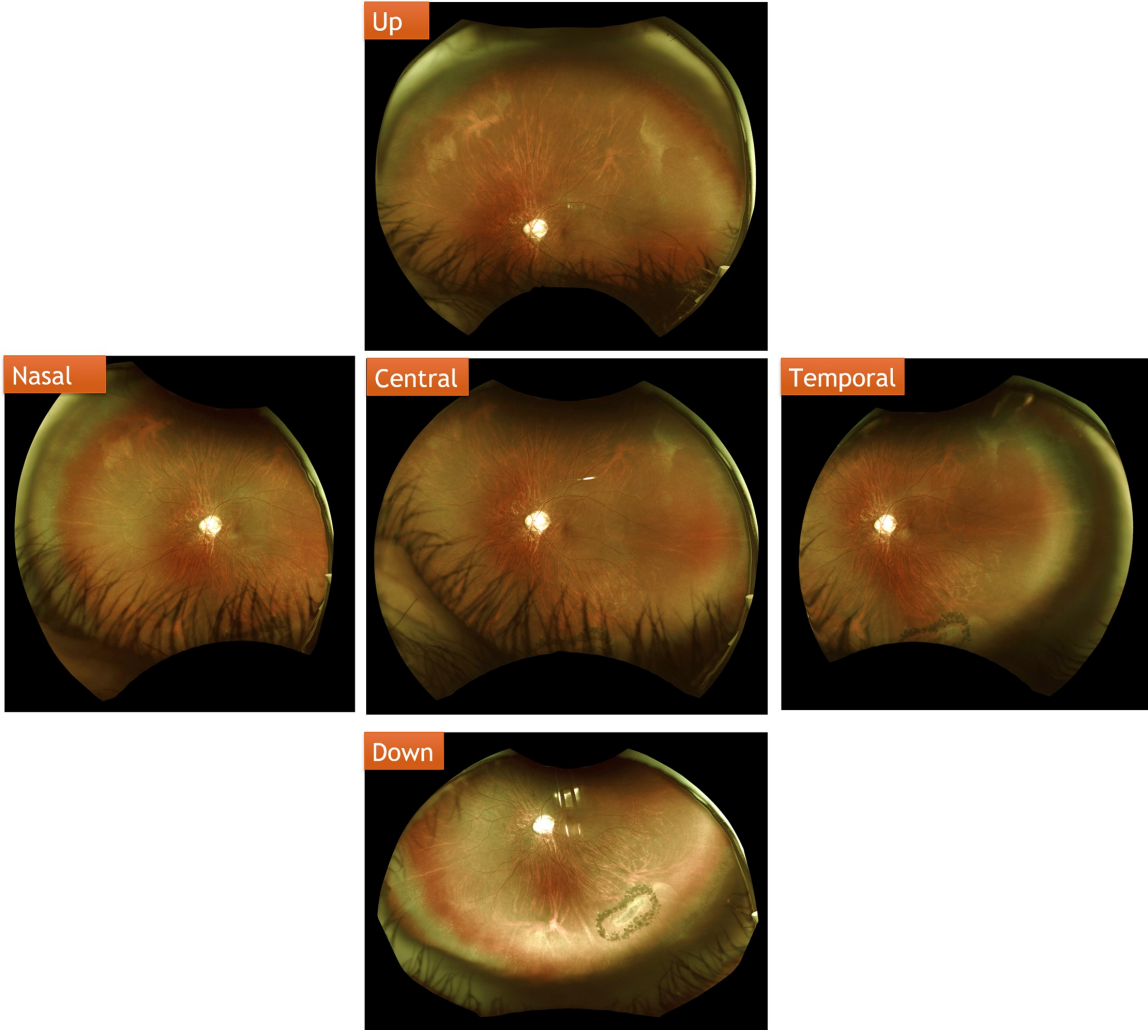
Central and four gaze-steered UWF images. Examples of the central image and four gaze-steered images (up, down, temporal, and nasal) taken during the UWF imaging examination.

**Fig 2:**
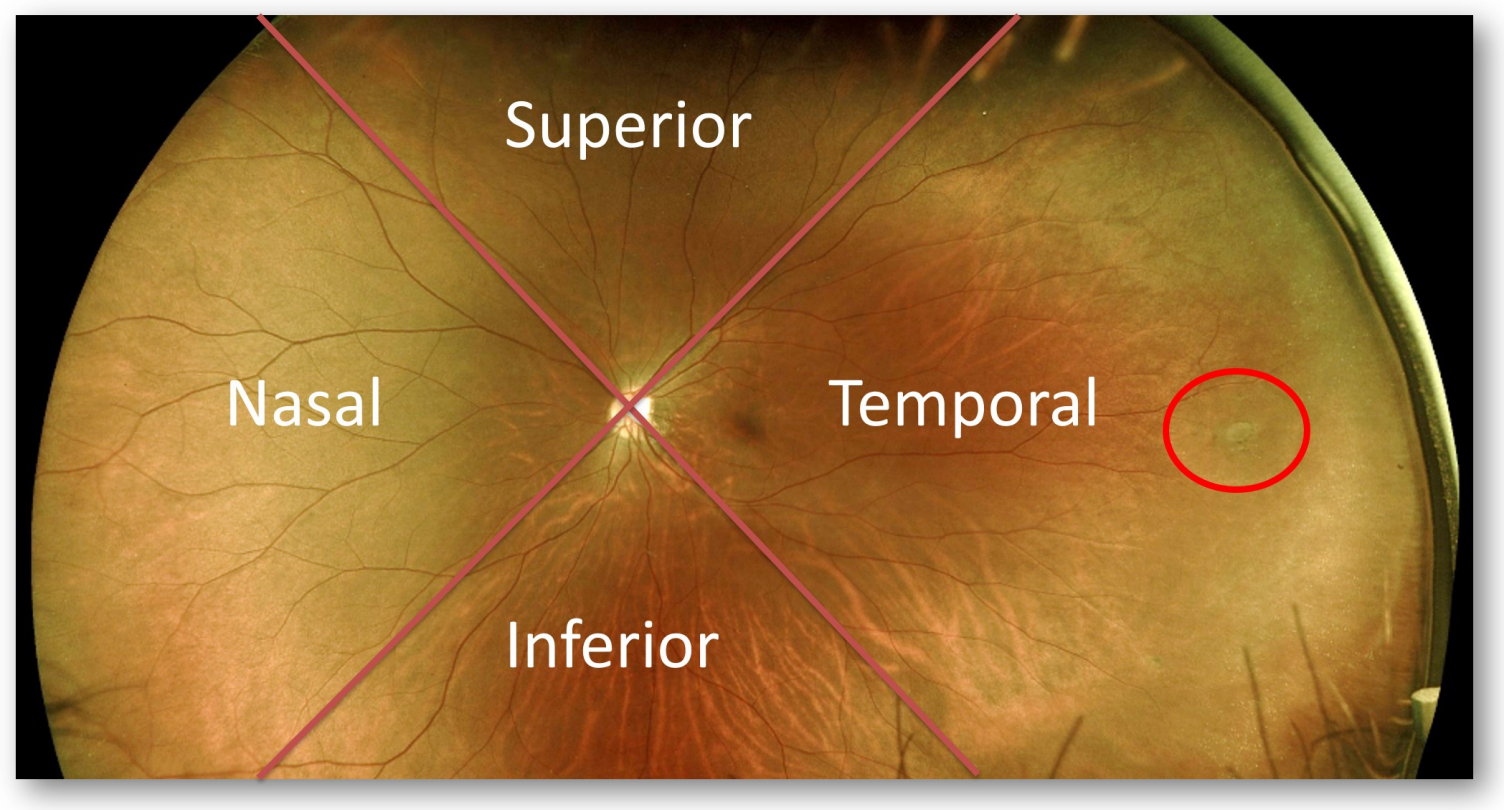
Four fundus quadrants and identification of retinal breaks. We divided the fundus into the superior, inferior, temporal, and nasal quadrants, with the optic disc at the center. We then counted the retinal breaks (red circle) of the total fundus and the four quadrants.

A central image and four gaze-steered images (up, down, temporal, and nasal) were taken during the UWF imaging examination (Fig 1). On the resulting images of both examinations, we divided the fundus into four quadrants–the superior, inferior, temporal, and nasal quadrants–with the optic disc at the center (Fig 2). We compared the numbers of retinal breaks that was found by indirect ophthalmoscopy examinations with those detected by UWF imaging (Fig 2) in the total fundus and in the four quadrants. The results of UWF imaging examination were interpreted by randomly assigned one of these two attending ophthalmologists. On comparing the number and location of retinal breaks as assessed using indirect ophthalmoscopy by qualified attending ophthalmologists and the UWF imaging examination, the results were the same between the two groups (S1 Table).

### Statistical analysis

Statistical analysis was performed using IBM-SPSS for Windows software, version 22 (SPSS Inc., Chicago, IL, USA). Due to a small sample and because we wanted to study if there are non-random associations between the two categorical variables (a retinal break can or cannot be detected by UWF imaging or indirect ophthalmoscopy), Fisher’s exact test was used to compare retinal break identification ability between UWF imaging and indirect ophthalmoscopy within the junior and senior resident groups. A p-value smaller than 0.05 was considered to be statistically significant.

## Results

**Fig 3:**
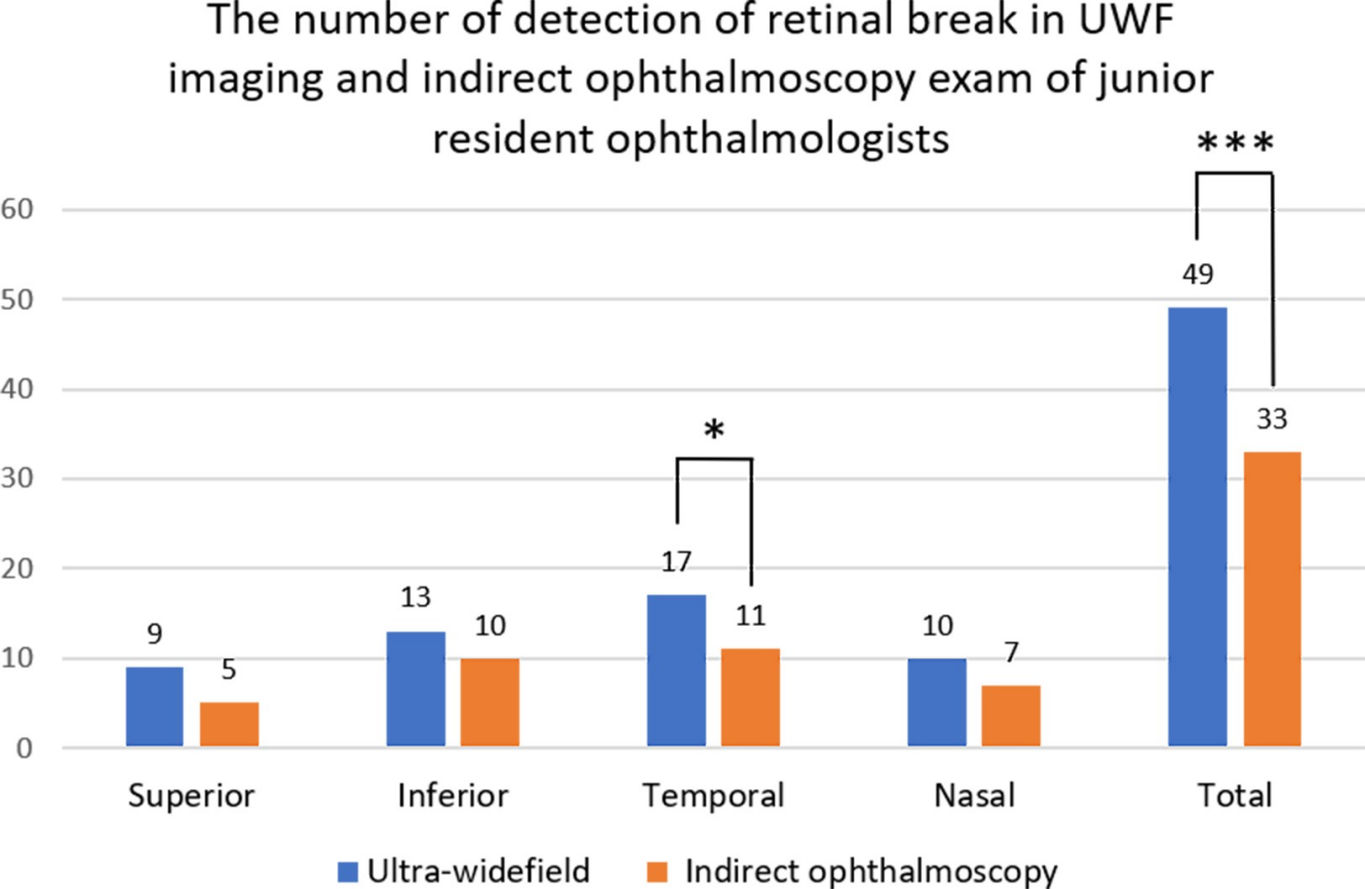
Number of retinal breaks detected by indirect ophthalmoscopy and UWF imaging examinations by junior resident ophthalmologists. For the indirect ophthalmoscopy examination, a total of 33 retinal breaks were detected in the 31 eyes. There were 5, 10, 11, and 7 retinal breaks detected in the superior, inferior, temporal, and nasal quadrants, respectively. For the UWF imaging, a total of 49 retinal breaks were detected in the 31 eyes, and there were 9, 13, 17, and 10 retinal breaks detected in the superior, inferior, temporal, and nasal quadrants, respectively. *P < .05, ** *P* < .01, ***P < .001, Fisher’s exact test.

Data from 53 eyes of 44 patients (22 male, 22 female) with retinal breaks were collected. The mean age of patients was 52.5 years (range, 27–71 years). The lens status was phakic, pseudophakic, and aphakic in 43, 10, and 0 eyes, respectively. We divided patients into two groups–the group in which examinations were conducted by junior residents and the group in which examinations were conducted by senior residents. For the group examined by junior residents, 31 eyes of 25 patients (13 male, 12 female) were included. The mean age of these patients was 52.3 years (range, 31–71 years). The lens status was phakic, pseudophakic, and aphakic in 26, 5, and 0 eyes, respectively. For the indirect ophthalmoscopy examination made by junior residents, a total of 33 retinal breaks were detected in the 31 eyes; 5, 10, 11, and 7 retinal breaks were detected in the superior, inferior, temporal, and nasal quadrants, respectively. For the UWF imaging examination, a total of 49 retinal breaks were detected in the 31 eyes, and there were 9, 13, 17, and 10 retinal breaks detected in the superior, inferior, temporal, and nasal quadrants, respectively (Fig 3). Every break detected by indirect ophthalmoscopy could be detected by UWF imaging, but some of the breaks detected by UWF imaging could not be detected by indirect ophthalmoscopy. Overall, UWF imaging could detect more breaks in all four quadrants. Using Fisher’s exact test, we found that UWF imaging was significantly better at detecting retinal breaks of the total fundus compared to indirect ophthalmoscopy (p < 0.001). However, when comparing the detection ability of retinal breaks in the four quadrants, the only significant difference between the two methods was in the temporal quadrant (p = 0.018), with no significant difference in the ability to detect retinal breaks in the superior (p = 0.082), inferior (p = 0.22), or nasal (p = 0.21) quadrants.

**Fig 4:**
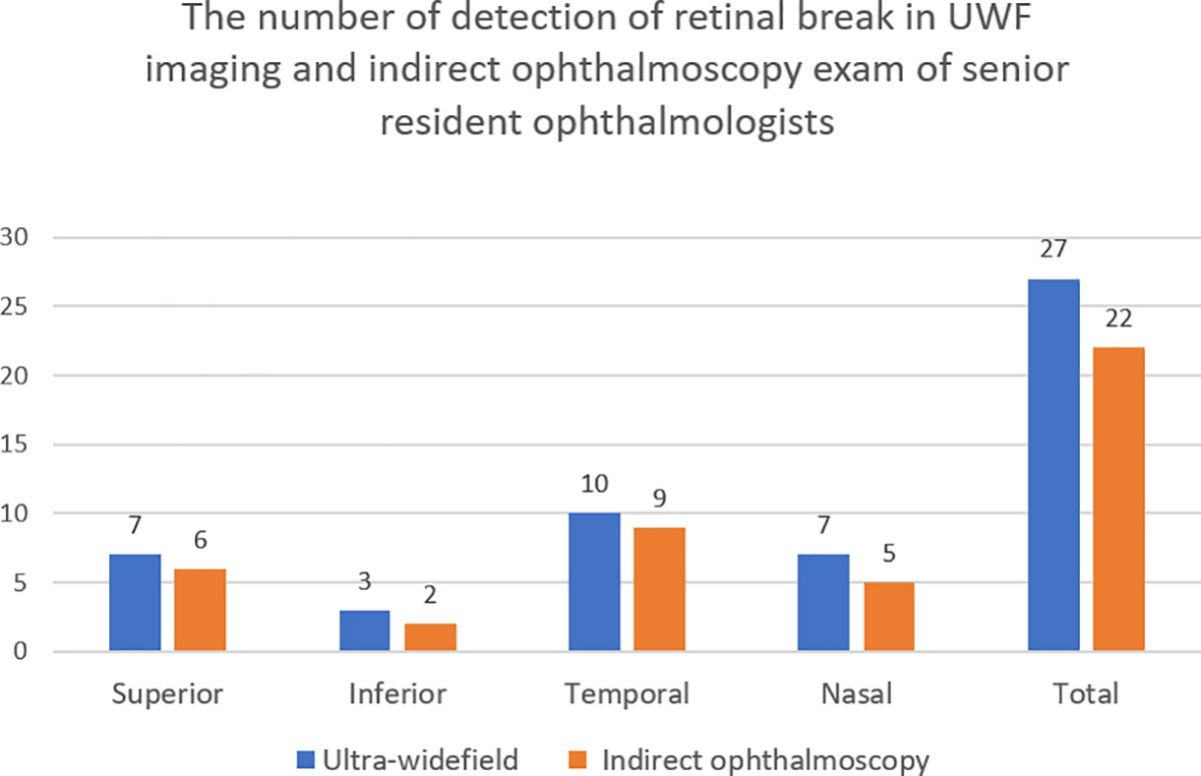
Number of retinal breaks detected by indirect ophthalmoscopy and UWF imaging examinations performed by senior resident ophthalmologists. For the indirect ophthalmoscopy examination, a total of 22 retinal breaks were detected in the 22 eyes; there were 6, 2, 9, and 5 retinal breaks detected in the superior, inferior, temporal, and nasal quadrants, respectively. For the UWF imaging, a total of 27 retinal breaks were detected in the 22 eyes; there were 7, 3, 10, and 7 retinal breaks detected in the superior, inferior, temporal, and nasal quadrants, respectively.

For the group examined by senior residents, 22 eyes of 19 patients (9 male, 10 female) were included. The mean age of patients was 52.7 years (range, 27–68 years). The lens status was phakic, pseudophakic, and aphakic in 17, 5, and 0 eyes, respectively. For the indirect ophthalmoscopy examination made by senior residents, a total of 22 retinal breaks were detected in the 22 eyes, with 6, 2, 9, and 5 retinal breaks detected in the superior, inferior, temporal, and nasal quadrants, respectively. For the UWF imaging examination, a total of 27 retinal breaks were detected in the 22 eyes, with 7, 3, 10, and 7 retinal breaks detected in the superior, inferior, temporal, and nasal quadrants, respectively (Fig 4). As observed in the junior resident group, every break detected by indirect ophthalmoscopy could be detected by UWF imaging, but some of breaks detected by UWF imaging could not be detected by indirect ophthalmoscopy. UWF imaging could detect more breaks in all four quadrants. Using Fisher’s exact test, we found that there was no significant difference between UWF imaging and indirect ophthalmoscopy in their ability to detect retinal breaks of the total fundus (p = 0.051). However, this result shows a strong tendency of UWF imaging to yield better results than indirect ophthalmoscopy in detecting retinal breaks of the total fundus. Fisher’s exact test revealed no significant difference between the two methods in their ability to detect retinal breaks within the superior (p = 1.000), inferior (p = 1.000), temporal (p = 1.000), and nasal (p = 0.462) quadrants.

## Discussion

In this study, UWF imaging had a significantly better ability to detect retinal breaks of the total fundus and temporal quadrant compared to indirect ophthalmoscopy when performed by junior residents. Daniel et al. reported that the detection rate of retinal holes and tears in patients with rhegmatogenous retinal detachment is increased in nasal quadrants, but decreased in superior and inferior quadrants, when using UWF imaging to take the central image as compared to indirect ophthalmoscopy [18]. However, their study used only the central image, which includes 82% of the retina. By taking a central image and four gaze-steered images (up, down, temporal, and nasal) when performing a UWF imaging examination, up to 97% of the retina is visible [17]. Additionally, their study did not compare senior residents’ performance to that of junior residents. We compared the junior residents’ ability to detect retinal breaks using UWF imaging examination with central and four gaze-steered images or indirect ophthalmoscopy. We found that UWF imaging examination has a significantly better detection rate in the total fundus and temporal quadrant than that of indirect ophthalmoscopy when performed by junior ophthalmology residents. We also found a trend towards a better detection rate using UWF imaging in the superior, inferior, and nasal quadrants.

The better retinal break detection rate in the total fundus for UWF imaging compared to indirect ophthalmoscopy when examined by junior residents could be explained in two ways. First, UWF imaging examinations allow up to 97% of the retina to be visualized, by taking a central image and four gaze-steered images. Therefore, it can be used to visualize the peripheral retinal break, which is easily missed. Second, junior resident ophthalmologists did not have proficient skills or profound experience with the indirect ophthalmoscopy examination. In contrast, senior resident ophthalmologists have proficient skills and profound experience with the indirect ophthalmoscopy examination. Therefore, there was no significant difference in the ability to detect retinal breaks in the total fundus or each of the four quadrants with UWF imaging or indirect ophthalmoscopy when performed by senior residents. Our results indicate that UWF may be a valuable tool for ophthalmology residents to gain experience and proficiency in the examination of retinas and the detection of breaks.

It was interesting to find that UWF imaging also had a better retinal break detection rate in the temporal quadrant than that of indirect ophthalmoscopy when examined by the junior residents. While our results may seem at odds with other examples which showed a better retinal break detection rate in the nasal quadrant from the literature [14], They can be explained because the temporal and nasal quadrants have a common feature, which is that they have no interference from the eyelids and eyelashes. Therefore, UWF imaging may provide a better visible view, resulting in a better detection rate of retinal breaks in these areas. For the superior and inferior quadrants, the blockage of the peripheral retina image by the upper and lower eyelids or eyelashes is sometimes obvious, which could explain why UWF imaging did not have a significantly better detection rate of retinal breaks than indirect ophthalmoscopy in these areas.

Our study has some limitations. First, although using indirect ophthalmoscopy with scleral depression is considered as the gold standard for the diagnosis of retinal breaks, our indirect ophthalmoscopy examination did not use scleral depression because it can be painful for conscious patients. However, this could lower the detection rate for retinal breaks in peripheral areas. Second, due to the small sample size, this study might be underpowered. Future large-scale studies are needed to validate our results. Third, we did not have a large enough sample size to perform a subgroup analysis between the phakic, pseudophakic, or different cataract severities patients, in whom the ability to detect retinal breaks might differ between UWF imaging and indirect ophthalmoscopy examinations.

## Conclusion

UWF imaging using a central image and four gaze-steered images is a useful adjunct tool for the detection of retinal breaks in the superior, inferior, temporal, and nasal quadrants, especially for junior residents. For this group of trainees, UWF imaging gave them a significantly better ability to detect retinal breaks of the total fundus and temporal quadrant than that of indirect ophthalmoscopy. Therefore, UWF imaging can be considered as a useful, adjunctive tool in training of residents for retinal break detection. Additionally, it could be a useful method for teaching indirect ophthalmoscopy examination to junior residents.

## Supporting information

S1 Table The number of retinal breaks detected by indirect ophthalmoscopy performed by qualified attending ophthalmologists and by UWF imaging examination. (DOCX)

S1 Dataset (XLSX)
