# Supplementary material for: Ultra-widefield retinal imaging for adjunctive resident training in retinal break detection
Source: PLoS One. 2021 Jun 23;16(6):e0253227. doi: 10.1371/journal.pone.0253227 (PMC8221479; doi:10.1371/journal.pone.0253227)
Supplement: S1 Table — (DOCX) [file pone.0253227.s001.docx]

**S1 Table.** The number of retinal breaks detected by indirect ophthalmoscopy performed by qualified attending ophthalmologists and by UWF imaging examination.

|  | The number of retinal breaks detected | | | | | | | | | |
| --- | --- | --- | --- | --- | --- | --- | --- | --- | --- | --- |
| Group | Junior student group | | | | | Senior student group | | | | |
| Location | superior | inferior | temporal | nasal | Total | superior | inferior | temporal | nasal | Total |
| Indirect ophthalmoscopy examination by qualified attending ophthalmologists | 9 | 13 | 17 | 10 | 49 | 7 | 3 | 10 | 7 | 27 |
| UWF imaging examination | 9 | 13 | 17 | 10 | 49 | 7 | 3 | 10 | 7 | 27 |
